# Dysbiosis of the salivary microbiota in pediatric-onset primary sclerosing cholangitis and its potential as a biomarker

**DOI:** 10.1038/s41598-018-23870-w

**Published:** 2018-04-03

**Authors:** Kentaro Iwasawa, Wataru Suda, Tomoyuki Tsunoda, Manari Oikawa-Kawamoto, Shuichiro Umetsu, Lena Takayasu, Ayano Inui, Tomoo Fujisawa, Hidetoshi Morita, Tsuyoshi Sogo, Masahira Hattori

**Affiliations:** 1 Department of Pediatric Hepatology and Gastroenterology, Saiseikai Yokohamashi Tobu Hospital, Kanagawa, 230-8765 Japan; 2 Department of Pediatrics, Yokohama Minami Kyousai Hospital, Kanagawa, 236-0037 Japan; 3 Department of Pediatrics, Graduate School of Medicine, Yokohama City University, Kanagawa, 236-0004 Japan; 4 Laboratory for Microbiome Sciences, RIKEN Center for Integrative Medical Sciences, Kanagawa, 230-0045 Japan; 5 Department of Microbiology and Immunology, Keio University School of Medicine, Tokyo, 108-8345 Japan; 6 Graduate School of Frontier Sciences, The University of Tokyo, Chiba, 277-8561 Japan; 7 Department of Gastroenterology and Hepatology, Tokyo Medical and Dental University, Tokyo, 113-8510 Japan; 8 Children’s Center for Health and Development, Saiseikai Yokohamashi Tobu Hospital, Kanagawa, 230-8765 Japan; 9 Graduate School of Environmental and Life Science, Okayama University, Okayama, 700-0082 Japan; 10 Graduate School of Advanced Science and Engineering, Waseda University, Tokyo, 169-8555 Japan

## Abstract

Primary sclerosing cholangitis (PSC) is a liver disease known for its frequent concurrence with inflammatory bowel disease. Dysbiosis of the gut microbiota in PSC was reported in several studies, but the microbiological features of the salivary microbiota in PSC have not been established. Here we compared the salivary microbial communities of 24 pediatric-onset PSC patients, 16 age-matched ulcerative colitis (UC) patients, and 24 healthy controls (HCs) by analyzing the bacterial 16S rRNA gene sequence data. The species-richness (α-diversity) showed no significant between-group differences, whereas the overall salivary microbiota structure (β-diversity) showed significant differences among the three groups. Taxonomic assignment revealed that the PSC salivary microbiota were characterized by significant decreases in the abundance of *Rothia* and *Haemophilus* compared to the HC group, and significantly decreased *Haemophilus* and increased *Oribacterium* compared to the UC group. By combining the genera selected by the random forest algorithm in machine learning, followed by confirmation with 10-fold cross-validation, we were able to distinguish the PSC group from the HC group with the area under the curve (AUC) of 0.7423, and from the UC group with the AUC of 0.8756. Our results indicate the potential of salivary microbiota as biomarkers for a noninvasive diagnosis of PSC.

## Introduction

Primary sclerosing cholangitis (PSC) is a liver disease characterized by multiple stenoses due to chronic inflammation and fibrosis in the intrahepatic and extrahepatic biliary system. The disease eventually progresses to end-stage liver disease, resulting in poor prognoses^1^. PSC is also known for its high association with inflammatory bowel disease (IBD), mainly ulcerative colitis (UC), with a prevalence of 70–80% in adults^1^, and with the same proportion or an even higher proportion in children^2–4^. Conversely, as many as 8.1% of IBD patients develop PSC^5^. It was also reported that PSC-like bile duct lesions occur frequently even with normal biochemical profiles in children with IBD^6^.

In light of this frequent concurrence of PSC and IBD, the diagnosis of either of these diseases should consider the concurrence of the other disease. Cholangiography is mandatory for the diagnosis of PSC, and endoscopic retrograde cholangiopancreatography (ERCP) has been used for the diagnosis of PSC in adults and children^7^. However, ERCP is associated with complications such as pancreatitis. To avoid complications, magnetic resonance cholangiopancreatography (MRCP) has been proposed as an alternative method for the diagnosis of PSC, but its sensitivity is approximately 81–86% in children, with a lower resolution in younger patients and the need for sedation in some cases^8,9^. Thus, additional noninvasive diagnostic biomarkers are desirable for the evaluation of the presence of PSC.

In pediatric and adult PSC patients, the oral administration of antibiotics was reported to decrease the serum levels of alanine aminotransferase (ALT) and γ-glutamyltranspeptidase (GGT), with histological improvement^10–14^, implying that gastrointestinal inflammation and indigenous microbes are associated with the pathogenesis and prognosis of PSC, and that early intervention may also produce better prognoses. The gut microbiota of adult PSC patients were recently intensively evaluated by using 16S rRNA gene sequences^15–23^. Those studies indicated the enrichment of particular bacterial genera including *Veillonella*^19,23^*, Enterococcus*^20,23^*, Lactobacillus*^20^, *Ruminococcus*^22^*, Streptococcus*^23^, *Rothia*^23^, and *Fusobacterium*^20,22^ in the feces of PSC patients. We also reported the enrichment of several species, some belonging to these genera, in a Japanese pediatric-onset PSC cohort^2^.

In addition to close associations between dysbiosis of the gut microbiota and diseases^24^, several studies have described alterations of salivary microbiota in patients with various diseases. These include cirrhosis^25^, IBD^26,27^, pancreatic cancer^28,29^, lung cancer^30^, colorectal cancer^31^, IgA nephropathy^32^, celiac disease^33^, Behcet’s disease^34^, and rheumatoid arthritis^35^. The involvement of salivary microbes in these diseases is unclear, but a correlation between the gut and oral microbiota was suggested^27,36^. In addition, changes in the salivary microbiota in systemic diseases may be a resource of microbial biomarkers specific to the diseases, which could contribute to the development of simple and noninvasive diagnoses based on microbial profiling with saliva samples.

To date, there has been no report on the salivary microbiota of PSC patients. As the initial step in the exploration of potential biomarkers for the noninvasive diagnosis of PSC, we performed a bacterial 16S rRNA gene sequence analysis to characterize the salivary microbiota of pediatric-onset PSC patients in a comparison of their microbiota with those of pediatric-onset UC patients and healthy controls (HCs).

## Results

### Patients, clinical data, and healthy subjects

We recruited 24 PSC and 16 UC patients without PSC whose age of onset was <18 years from Saiseikai Yokohamashi Tobu Hospital (Kanagawa, Japan) between May 2013 and October 2015, and healthy volunteers without any symptoms (Table 1). We collected salivary samples from 24 patients with PSC (the PSC group, median age 12.5 years), 16 patients with UC without PSC (the UC group, median age 12.5 years), and 24 healthy controls (the HC group, median age 11.5 years).

**Table 1:** Demographics and characteristics of the PSC, UC, and HC subjects used.

	PSC	UC	HC	*p*-value
Number of patients	24	16	24	
Male, n (%)	16 (67)	7 (44)	10 (42)	0.172
Present age, yrs, median (IQR)	12.5 (9.5–17.5)	12.5 (11.5–15.5)	11.5 (5.5–16)	0.467
Age at onset, yrs, median (IQR)	6 (3–10)	11 (7.5–12.5)	—	**0.026**
Age at diagnosis, yrs, median (IQR)	9 (6–12)	12 (9–13)	—	0.161
PSC phenotype n (%)				
Large duct PSC	23 (96)	—	—	
Small duct PSC	1 (4)	—	—	
Overlap with autoimmune hepatitis	14 (58)	—	—	
Type of IBD, n (%)	24 (100)	16 (100)	—	1.000
UC	10 (42)	16 (100)	—	**<0.001**
IBD-U	14 (58)	0 (0)	—	**<0.001**
PUCAI score in UC patients, n (%)				
Remission (0–9)	7 (70)	8 (50)	—	0.428
Mild (10–30)	3 (30)	5 (31)	—	0.945
Moderate to severe (35–)	0 (0)	3 (19)	—	0.262
Biochemical data, median (IQR)				
Platelets, 10^9^/μL	274.5 (240.5–341.5)	270.5 (240.5–366.0)	—	0.801
Albumin, g/dL	4.45 (4.1–4.75)	4.5 (3.85–4.6)	—	0.617
AST, IU/L	55 (24.5–84)	22 (19.5–24)	—	**<0.001**
ALT, IU/L	42.5 (15.5–98.5)	13.5 (12–20.5)	—	**0.003**
GGT, IU/L	60.5 (12.5–131)	12 (10–17)	—	**<0.001**
APRI	0.42 (0.22–1.25)	0.22 (0.17–0.25)	—	**0.002**
Medication n (%)				
UDCA	20(83)	0 (0)	—	**<0.001**
SASP	13 (54)	1 (6)	—	**0.002**
Mesalazine	5 (21)	14 (88)	—	**<0.001**
Immunosuppresive	9 (38)	10 (63)	—	0.220
Steroids	6 (25)	2 (13)	—	0.439
Probiotics	5 (21)	6 (38)	—	0.277

ALT, alanine aminotransferase; AST, asparate aminotransferase; APRI, AST to platelet ratio index; GGT, γ-glutamyl transferase; IBD, inflammatory bowel disease; IBD-U, inflammatory bowel disease unclassified; IQR, interquartile range; PUCAI, pediatric ulcerative colitis activity index; SASP, salazosulfapyridine; UDCA, ursodeoxycholic acid.

The diagnosis of PSC was made based on characteristic bile duct changes with multifocal strictures and segmental dilatation on cholangiography, clinical presentation, and the cholestatic biochemical profile, and no evidence of secondary sclerosing cholangitis^7^. The diagnosis of UC was made using the revised Porto criteria^37^.

This study was approved by the ethical committees of Saiseikai Yokohamashi Tobu Hospital, Azabu University, Waseda University, and the University of Tokyo, and signed informed consent was obtained from all subjects who provided specimens.

### The collection of 16S rRNA gene sequences of salivary microbiota of the three subject groups

We obtained a total of 458,021 high-quality 16S reads from the three groups by using the Roche 454 platform (see Methods, Supplementary Table S1). Of them, we randomly selected 3,000 reads per sample, accounting for a total of 192,000 reads from 64 samples, and analyzed them to minimize the overestimation of the species richness in the clustering due to intrinsic sequencing error^38^. The Good’s coverage index^39,40^ of the 3,000 reads per sample was 0.971, indicating a high coverage degree which was sufficient for the analysis.

### Relationship between medications and phenotypes of the PSC patients with the salivary microbial composition

Some of the PSC and UC patients had been treated with various medications (Table 1). Since we observed a significant alteration of the gut microbiota of PSC patients due to salazosulfapyridine (SASP) treatment^2^, we first evaluated the effect of medications on the salivary microbiota of the patients by comparing the data between those with and without medication use. The comparative analyses of the PSC samples with and without SASP treatment (Supplementary Table S2) revealed no significant difference between the two groups in the observed and Chao 1-estimated operational taxonomic units (OTU) number or in Shannon’s diversity index (Supplementary Fig. S1A). In addition, the weighted and unweighted UniFrac distance analyses also showed no significant difference between the PSC samples with and without SASP treatment (Supplementary Fig. S1B,C).

The permutational multivariate analysis of variance (PERMANOVA) also confirmed no significant difference between the PSC samples with and without SASP treatment, or between the PSC patients treated and untreated with ursodeoxycholic acid (UDCA), mesalazine, and probiotics respectively (Supplementary Table S3). In addition, the overlap with AIH and the concurrence of UC or IBD-U in the PSC patients showed no significant differences (Supplementary Table S3). These data suggested that the administration of these medications and the phenotype of PSC did not largely affect the salivary microbiota of the PSC patients. We therefore further analyzed all of the samples regardless of their use or non-use of medications and the phenotype of PSC.

### Comparison of the salivary microbiota of the PSC, UC, and HC samples

**Figure 1:**
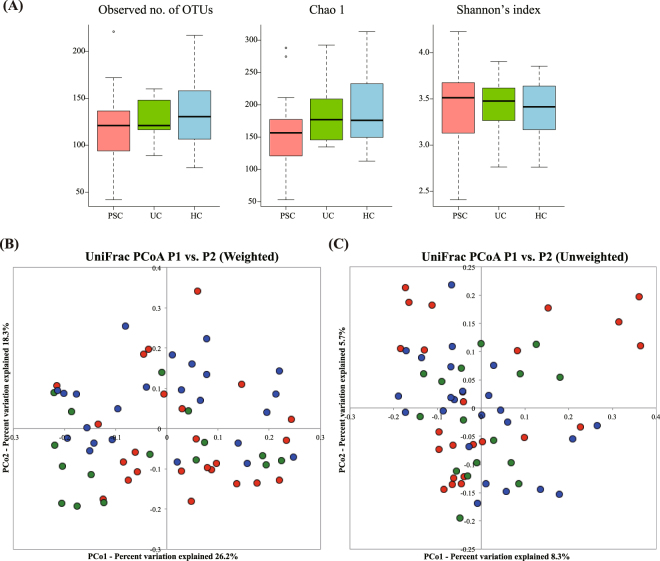
Comparison of the salivary microbiota of the PSC, UC, and HC subjects. Samples from 24 PSC (red), 16 UC (green) and 24 HC (blue) subjects are shown. (**A**) The observed and the Chao 1-estimated OTU numbers, and the Shannon’s index of salivary microbiota from the three groups. (**B**) Weighted UniFrac-PCoA and (**C**) unweighted UniFrac-PCoA of salivary microbiota from the three groups. OTU, operational taxonomic unit; PCoA, principal coordinate analysis.

Our comparative analysis of the PSC, UC and HC samples revealed that the observed and Chao 1-estimated OTU numbers of the PSC group tended to be lower than those of the UC and HC groups, without significant differences (Fig. 1A). Shannon’s index also showed no significant differences in bacterial diversity among the three groups (Fig. 1A).

The principal coordinate analysis (PCoA) plot based on the weighted UniFrac distance metric showed that many of the PSC and UC samples segregated from the HC samples, suggesting that the salivary microbiota structure of both the PSC and UC groups differed from that of the HC group (Fig. 1B). The PERMANOVA further revealed significant differences between the PSC and UC groups in the weighted UniFrac metric but not in the unweighted UniFrac metric (Table 2). Collectively, these data suggested that the salivary microbiota of the PSC group exhibited microbial dysbiosis without a large alteration of the species richness, but that changes in the abundance of the shared species among the three groups largely contributed to the overall structural differences in salivary microbiota across the three groups.

**Table 2:** Permutational multivariate analysis of variance in salivary microbiota samples among the PSC, UC and HC groups.

Category	No. of subjects	Weighted UniFrac			Unweighted UniFrac		
		R^2^	*p*-value	Adjusted *p*-value	R^2^	*p*-value	Adjusted *p*-value
PSC vs. UC	PSC:24 UC:16	0.04912	**0.04496**	**0.04496**	0.03222	0.06893	0.1544
PSC vs. HC	PSC:24 HC:24	0.04403	**0.03097**	**0.04496**	0.02567	0.1029	0.1544
UC vs. HC	UC:16 HC:24	0.0638	**0.01800**	**0.04496**	0.02723	0.2847	0.2847

The adjusted *p*-values were adjusted for multiple testing by Benjamin-Hochberg procedure. Significant p-values are in bold.

We then taxonomically assigned the OTUs at the phylum and family levels according to a similarity search against the public databases. The taxonomic assignment indicated that the major phyla in the three groups were Firmicutes, Bacteroidetes, Proteobacteria, Actinobacteria, Fusobacteria, and TM7. The phylum-level comparison showed that the abundance of Bacteroidetes and Actinobacteria in the PSC group and the abundance of Firmicutes and TM7 in the UC group differed significantly from that in the HC group (Supplementary Fig. S2A). At the family level, of the 14 families with the relative mean abundance of >0.5%, accounting for 93.1% of the total abundance, the abundance of *Streptococcaceae, Pasteurellaceae*, and *Lachnospiraceae* showed significant differences among the three groups. Of these three families, a lower *Pasteurellaceae* abundance and a higher *Lachnospiraceae* abundance were observed in the PSC group compared to both the UC and HC groups (Supplementary Fig. S2B). The abundance of *Streptococcaceae* was significantly lower in only the UC group compared to the HC group (Supplementary Fig. S2B).

**Figure 2:**
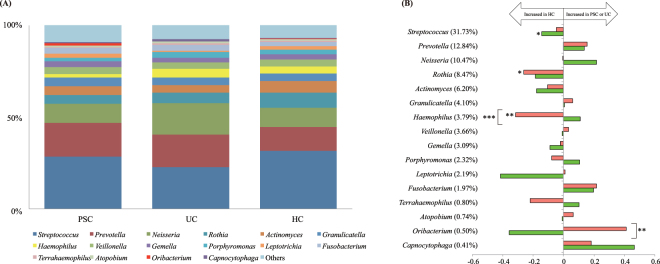
Comparison of the bacterial composition among the PSC, UC, and HC groups. (**A**) The average microbial abundance of the 16 dominant genera with the relative mean abundances of >0.5% are represented. All others are summed into the category “others.” (**B**) Fold-change of the 16 dominant genera in PSC/HC and UC/HC comparisons, which are calculated by dividing the mean relative abundance (%) of each genus in the PSC and UC groups by that in the HC group, respectively. The mean abundance (%) in the HCs is shown in parentheses. Horizontal axis: The fold-change displayed in log10. Horizontal bars: The fold-change between PSC and HC (red) and between UC and HC (green). *p < 0.05, **p < 0.01, and ***p < 0.001 based on the Kruskal-Wallis test followed by the Steel-Dwass test for multiple comparisons.

The taxonomic assignment at the genus level identified a total of 114 bacterial genera, of which we further evaluated the major 16 genera with the relative mean abundances of >0.5%, accounting for 92.2% of the total abundance (Fig. 2). As shown in Fig. 2B, the abundances of *Rothia* and *Haemophilus* were significantly lower in the PSC group compared to the HC group (p = 0.0454 and 0.0036, respectively). Moreover, the abundance of *Haemophilus* was also significantly lower in the PSC group than that in the UC group (p = 0.0007). Conversely, the abundance of *Oribacterium* was significantly higher in the PSC group compared to the UC group (p = 0.0036). Additionally, the abundance of *Streptococcus* was significantly lower in the UC group compared to the HC group (p = 0.0159).

**Figure 3:**
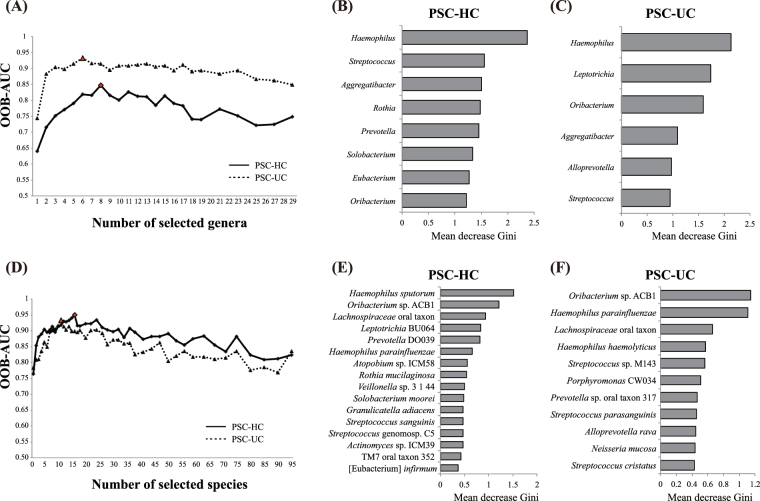
Random forest (RF) analysis of the salivary microbiota in the three subject groups. RF is performed at the genus-level (**A**–**C**) and at the species level (**D**–**F**). The best RF model with the highest AUC is indicated by a red circle in the PSC-HC comparison (**A**), and a red triangle in the PSC-UC comparison (**D**). The taxa selected in the best RF model are shown in (**B**,**E**) for the PSC-HC comparison and in (**C**,**F**) for the PSC-UC comparison. The bars indicate the mean decrease Gini, and the colors indicate the fold-change, which were calculated by dividing the mean relative abundance (%) of each genus and species in the PSC by that in the HC group (**B**,**E**) and the UC group (**C**,**F**), respectively. PCA, principal component analysis.

To explore the taxa contributing to differences between any pair of the three groups, we generated random forest (RF) models using the AUC-RF package^41^. All receiver operating characteristic (ROC) curves were based on the out-of-bag (OOB) error rates in the RF models. We used the area under the curve (AUC) of these ROC curves to find the combination of multiple taxa contributing most to the discrimination of the three groups from the 29 abundant genera with relative mean abundances of >0.1%. The best model was selected according to the best OOB-AUC, which was observed for eight and six genera between the PSC and HC groups, and the PSC and UC groups, respectively (Fig. 3A).

Among these selected genera, four genera (*Haemophilus*, *Streptococcus*, *Aggregatibacter* and *Oribacterium*) were the common contributors to distinguish the PSC from both the HC and UC groups (Fig. 3B,C and Supplementary Fig. S3A). Four genera (*Rothia*, *Prevotella*, *Solobacterium*, and *Eubacterium*) contributed to distinguishing the PSC from the HC group (Fig. 3B, Supplementary Fig. S3B), and the two genera *Leptotrichia* and *Alloprevotella* contributed to distinguishing the PSC from the UC group (Fig. 3C, Supplementary Fig. S3C). These results were further confirmed by evaluating the mean AUC of 10-fold cross-validation repeated 20 times, which showed a mean AUC of 0.7423 between the PSC and HC groups, and 0.8756 between the PSC and UC groups (Table 3).

**Table 3:** The mean area under the curve (AUC) of salivary microbiota samples among the PSC, UC, and HC groups.

Category	AUC	
	Genera	Species
PSC vs. UC	0.8756	0.7626
PSC vs. HC	0.7423	0.8011
UC vs. HC	0.6452	0.5314

The mean AUCs of the 10-fold cross-validation process repeated 20 times using the best RF model are shown.

The principal component analysis (PCA) using the RF-selected genera more clearly distinguished the PSC from the HC and UC groups than the PCA using the 29 genera with the mean abundance >0.1% (Supplementary Fig. S4). Similarly, a best mean AUC of 0.6452 was obtained between the UC and HC groups in the 10-fold cross-validation of the RF-selected genera (Table 3).

At a finer species level, we used 95 species with relative mean abundances of >0.1% to build the first RF. The best model was selected from the best OOB-AUC value, which was observed for 16 and 11 species between the PSC and HC groups, and between the PSC and UC groups, respectively (Fig. 3D). The three species *Oribacterium* sp. ACB1, *Lachnospiraceae* oral taxon 107, and *Haemophilus parainfluenzae* were the common species to distinguish PSC from both the HC and UC groups (Fig. 3E,F and Supplementary Fig. S5A). These species also exhibited significant differences in the relative abundance between the PSC and HC groups and between the PSC and UC groups (Supplementary Fig. S5A).

Of the 16 species, 13 (*Haemophilus sputorum*, *Leptotrichia* BU064, *Prevotella* DO039, *Atopobium* sp. ICM58, *Rothia mucilaginosa, Veillonella* sp. 3_1_44, *Solobacterium moorei*, *Granulicatella adiacens*, *Streptococcus sanguinis*, *Streptococcus* genomosp. C5, *Actinomyces* sp. ICM39, TM7 oral taxon 352 and [Eubacterium] *infirmum*) were the species distinguishing the PSC group from the HC group (Fig. 3E, Supplementary Fig. S5B). Of the 11 species, eight (*Haemophilus haemolyticus*, *Streptococcus* sp. M143, *Porphyromonas* CW034, *Prevotella* sp. oral taxon 317, *Streptococcus parasanguinis*, *Alloprevotella rava*, *Neisseria mucosa*, and *Streptococcus cristatus*) were the species distinguishing the PSC group from the UC group (Fig. 3F, Supplementary Fig. S5C).

The mean AUC of the 10-fold cross-validation repeated 20 times was 0.8011 between the PSC and HC groups and 0.7626 between the PSC and UC groups (Table 3). The PCA using the RF-selected species showed clear segregation between the PSC and HC groups, and between the PSC and UC groups (Supplementary Fig. S6). These specie-level contributors were further confirmed by a heatmap of the abundance of OTUs in the individuals (Supplementary Fig. S7). Similarly, a mean AUC of 0.5314 was obtained between the UC and HC groups in the 10-fold cross-validation (Table 3).

## Discussion

We compared salivary microbiota from age-matched pediatric-onset PSC patients, UC patients without PSC, and healthy individuals by using the enumerated 16S rRNA gene sequence data. The results indicated that compared to the healthy controls, the species richness of the PSC and UC salivary microbiota was not significantly changed. However, the overall structure of the PSC and UC salivary microbiota exhibited microbial dysbiosis compared to that of the healthy controls, and the observed dysbiosis was represented by significant changes in the abundance of several genera and species.

When we focused on the differences in the salivary microbiota between the PSC and UC groups, we observed significant differences in the abundance of two genera assigned to *Haemophilus* and *Oribacterium* and some species belonging to *Haemophilus*, *Oribacterium*, and *Lachnospiraceae* between the two groups. On the other hand, we observed previously that the abundance of *Faecalibacterium*, *Ruminococcus* and *Roseburia* genera was significantly different in the gut microbiota between the same PSC and UC individuals as those in the present study^2^. These data suggested almost no taxonomical similarity in the dysbiosis of salivary and gut microbiota between the PSC and UC groups, whereas larger numbers of species were different between them in the salivary microbiota than the gut microbiota. Interestingly, the PSC fecal microbiota were characterized by a significant enrichment of *S. parasanguinis*^2^, which was also one of the contributing species discriminating the PSC from the UC salivary microbiota in the present study (Fig. 3F), but a significant difference was not observed between them (Supplementary Fig. S5C).

The large differences observed between the PSC and UC salivary microbial communities prompted us to investigate the potential of the microbiota as novel biomarkers discriminating PSC and UC patients. The cross-validation using salivary microbiota data identified several genera and species efficiently distinguishing the PSC patients from the healthy controls, and from the UC patients (Fig. 3, Table 3). Some of these AUC values were substantially higher than those reported in a study in which a combination of several gut species distinguished PSC from healthy individuals with an AUC of 0.78, and from UC with an AUC of 0.82^19^. We thus anticipate that salivary microbes can also be used as diagnostic biomarkers distinguishing PSC from healthy individuals and UC patients, similarly to gut microbes. Notably, since all the PSC patients in our cohort were associated with IBD, the identified salivary microbial biomarkers may also be efficient for distinguishing IBD patients with PSC from those without PSC.

Bajaj *et al*. reported decreased abundances of the families *Lachnospiraceae* and *Ruminococcaceae* in the salivary microbiota of patients with liver cirrhosis compared to healthy controls^25^. In contrast, although PSC is also a disease that eventually progresses to liver cirrhosis, the abundance of *Lachnospiraceae* was significantly increased in our PSC salivary samples compared to the UC and healthy control samples. This opposite change in the abundance of the family *Lachnospiraceae* in salivary microbiota between the two diseases may be due largely to the increase in the genus *Oribacterium* belonging to the family *Lachnospiraceae* in the PSC group and the reduction of this genus in the UC group compared to the healthy controls. *Oribacterium* was also shown to be one of the RF-selected genera contributing to the discrimination of PSC from UC.

Additionally, at the species level, two species (*Lachnospiraceae* oral taxon 107 and *Oribacterium* sp. ACB1, both belonging to the family *Lachnospiraceae*) were markedly enriched in the present pediatric-onset PSC group compared to the UC and healthy control groups, and were shown to be the species contributing to the discrimination of the two groups. Similar to the pediatric-onset PSC patients analyzed here, the family *Lachnospiraceae* was increased in the salivary microbiota of children with celiac disease^33^. On the other hand, *Streptococcus sanguinis* was concurrently enriched in celiac disease patients^33^, whereas this species was significantly decreased in our PSC group (Fig. 3E, Supplementary Fig. S6).

*Oribacterium* was also enriched in the microbiota of the tongue coat in patients with liver cancer, in which *Oribacterium* and *Fusobacterium* gave AUCs of 0.8137 and 0.7749 for distinguishing patients with liver cancer from healthy subjects, respectively^42^. Since PSC was also shown to have a risk for the development of liver cancer^43^ and its salivary microbiota composition is similar to that of the tongue and other intraoral microbiota^44,45^, further studies are needed to clarify the differences in salivary and tongue microbiota between these two hepatic diseases.

Several papers have described the potential of salivary microbes as biomarkers for disease. For example, a combination of two salivary species identified as metagenomic linkage groups separated rheumatoid arthritis patients from healthy subjects with an AUC of 0.814^35^. In another study, a combination of two salivary species, *Neisseria elongata* and *Streptococcus mitis*, was reported to be the biomarker discriminating pancreatic cancer patients from healthy controls with an AUC of 0.90^28^. A combination of two salivary genera, *Capnocytophaga* and *Veillonella*, yielded AUCs of 0.86 and 0.80 in distinguishing squamous cell carcinoma and adenocarcinoma in patients with lung cancer from healthy controls, respectively^30^.

These previous studies and our present investigation consider the combination of more than two different genera or species to be candidates for microbial biomarkers. We note that none of the microbial combinations reported to date overlap between diseases, suggesting that physiological states in these systemic diseases have different influences on the salivary microbiota. The alteration of salivary microbial profile would thus be a potent indicator that is sensitive to the host’s physiological changes in systemic disease.

In the diagnoses of clinical samples, medical treatments such as the administration of antibiotics are an issue to be considered because such treatments sometimes strongly change the microbial community structure, regardless of disease. We use exclusively SASP for gastrointestinal lesions in PSC, based on several reports^46,47^, and we observed that SASP treatment significantly changed the gut microbiota due to an antibacterial agent generated by the metabolism of SASP in the gut^2^. Therefore, a high tolerance of salivary microbiota to medical treatments is desired for the development of accurate diagnostics using the microbiota as biomarkers. In the present study, no significant difference in the salivary microbiota was observed in the PCS and UC salivary samples treated with SASP and mesalazine, in agreement with the previous report demonstrating the high stability of salivary microbiota compared to gut microbiota under the exposure of antibiotics^48^.

One of the limitations in our study is the small sample size. Pediatric-onset PSC is a rare disease, and it is difficult to collect large numbers of samples. To address this problem, we used cross-validation based on the best RF model to identify and validate microbial species that discriminate two groups with high fidelity to minimize overfitting. This may be a powerful strategy for analyses in which the number of samples is limited. In addition, sampling time in a day may also be considered for the accurate diagnosis because of considerable changes in the salivary microbiota composition due to the circadian rhythm^44^.

Our present findings demonstrated the potential of salivary microbes as tools for the noninvasive diagnosis of pediatric-onset PSC. However, it remains unknown whether the microbial markers identified here are also globally applicable to adult PSC patients and to PSC patients with different IBD status, genetic backgrounds, geographic locations, and dietary styles.

## Methods

### Sample collection and DNA extraction

All subjects were instructed to provide fresh saliva in a test tube during the daytime on the day of sample collection. The subjects were antibiotic-free for ≥2 weeks prior to sampling, except for SASP in the PSC group. The subjects were prohibited from eating or drinking for 2 hr prior to sample collection, but there was no restriction regarding the type of food eaten before that period. The saliva samples were immediately sealed in an AnaeroPack-Anaero (a plastic bag containing a disposable oxygen-absorbing and carbon dioxide-generating agent; Mitsubishi Gas Chemical, Tokyo) and then transported on ice to the laboratory within 24 hr. At the laboratory, the saliva samples were suspended in 20% glycerol and phosphate-buffered saline (PBS), and then immediately frozen in liquid nitrogen and stored at −80 °C until further analysis^49^.

The isolation of bacterial DNA from the salivary samples was performed as described^26^. The 16S rRNA gene V1–V2 region was amplified by polymerase chain reaction (PCR) using the forward primer 27Fmod (5′-CCATCTCATCCCTGCGTGTCTCCGACTCAGNNNNNNNNNNagrgtttgatymtggctcag-3′), containing the 454 primer A and a unique 10-bp barcode sequence for each sample (indicated by Ns), and the reverse primer 338 R (5′-CCTATCCCCTGTGTGCCTTGGCAGTCTCAGtgctgcctcccgtaggagt-3′) containing the 454 primer B^38^. The PCR was performed using 50 μL of 1× Ex Taq PCR buffer composed of 10 mM Tris-HCl (pH 8.3), 50 mM KCl, and 1.5 mM MgCl_2_ in the presence of 250 μM dNTPs, 1 U Ex Taq polymerase (Takara Bio, Kyoto, Japan), forward and reverse primers (0.2 μM), and approximately 20 ng of template DNA.

Thermal cycling was performed in a 9700 PCR System (Life Technologies Japan, Tokyo) with the following cycling conditions: initial denaturation at 96 °C for 2 min, followed by 25 cycles of denaturation at 96 °C for 30 sec, annealing at 55 °C for 45 sec, and extension at 72 °C for 1 min; and final extension at 72 °C. PCR amplicons were purified using AMPure XP magnetic purification beads (Beckman Coulter, Brea, CA, USA) and quantified using the Quant-iT PicoGreen dsDNA Assay Kit (Life Technologies Japan). An equal amount of each PCR amplicon was mixed and subjected to sequencing with the 454 GS FLX Titanium platform (Roche Applied Science, Indianapolis, IN) according to the manufacturer’s instructions.

### Data processing of 16S rRNA sequences

We used an analysis pipeline for the processing of the 454 pyrosequencing data of the 16S rRNA gene V1–V2 region, as reported^38^. Briefly, after multiplexed sequencing of the 16S amplicons, sequences were assigned to samples on the basis of their barcode sequences. Reads with an average quality value <25, inexact matches to both universal primers, and possible chimeric reads, totally accounting for 43–44% of all reads, were removed (Supplementary Table S1). Among the high-quality reads, 3,000 reads per sample were randomly chosen and used for the analysis conducted in this study.

We then sorted the selected reads with the average quality value and grouped them into OTUs by clustering using the UCLUST algorithm with a 96% identity threshold^26^. Taxonomic assignments for each OTU were made by similarity searching against the publicly available 16S (RDP and CORE) and NCBI genome databases using the GLSEARCH program. For assignment at the phylum, family, genus, and species levels, the sequence similarity thresholds of 70%, 90%, 94% and 96% were respectively applied. All of the high-quality 16S V1–V2 sequences analyzed in this study were deposited into the DDBJ/GenBank/EMBL database (accession no. DRA005698).

### Data analysis

Comparisons of the categorical variables were done using the Chi-squared test, Fisher’s exact test, Mann-Whitney U-test, and Kruskal-Wallis test where appropriate. The UniFrac distance was used for the assessment of the dissimilarity (distance) between any pair of samples^50^. We performed a principal coordinate analysis (PCoA) to visualize the similarities or dissimilarities in the microbiome structure in the UniFrac analysis. We conducted a PERMANOVA to compare the overall microbiome structure, and the p-values were adjusted for multiple testing by the Benjamin-Hochberg procedure. We used the observed and Chao 1-estimated OTU numbers and Shannon’s index to evaluate the richness and diversity of the overall microbial community. The similarity of the relative abundance at the phylum, family, genus, and species levels was assessed using the Kruskal-Wallis test followed by the Steel-Dwass test for multiple comparisons.

Random forest (RF) models were generated using the AUC-RF^41^ package. All receiver operating characteristic (ROC) curves presented for RF models were based on the out-of-bag (OOB) error rates. The area under the curve (AUC) of these ROC curves was used to find the most discriminatory variables (genera or species). The first RF was build, using all the variables, which provides the initial ranking of the variables according to its importance according to the mean decrease Gini. In the subsequent steps, 5% of the less important variables according to the initial ranking were eliminated. The RF was built with the remaining variables, and the AUC based on OOB predictions (OOB-AUC) of the RF was computed in each model. The best model was selected with the best OOB-AUC value. The best RF model was further confirmed by evaluating the mean AUC of a 10-fold cross-validation repeated 20 times using the AUC-RF^41^ package. All of these analyses were performed using the R software program (v3.3.1). All methods were carried out in accordance with relevant guidelines and regulations.

## Electronic supplementary material

Supplementary Files
