# Supplementary material for: Dysbiosis of the salivary microbiota in pediatric-onset primary sclerosing cholangitis and its potential as a biomarker
Source: Sci Rep. 2018 Apr 3;8:5480. doi: 10.1038/s41598-018-23870-w (PMC5882660; doi:10.1038/s41598-018-23870-w)
Supplement: Supplementary file 1 — Supplementary Files [file 41598_2018_23870_MOESM1_ESM.pdf]

Dysbiosis of the salivary microbiota in pediatric-onset primary sclerosing cholangitis and its potential as a biomarker

Kentaro Iwasawa[1–3*], Wataru Suda[4–6], Tomoyuki Tsunoda[7], Manari Oikawa-Kawamoto[1,8], Shuichiro Umetsu[1], Lena Takayasu[4,6], Ayano Inui[1], Tomoo Fujisawa[1], Hidetoshi Morita[9], Tsuyoshi Sogo[1†], and Masahira Hattori[4,6,10†]

[1]Department of Pediatric Hepatology and Gastroenterology, Saiseikai Yokohamashi Tobu Hospital, Kanagawa, 230-8765, Japan

[2]Department of Pediatrics, Yokohama Minami Kyousai Hospital, Kanagawa, 236-0037, Japan

[3]Department of Pediatrics, Graduate School of Medicine, Yokohama City University, Kanagawa, 236-0004, Japan

[4]Laboratory for Microbiome Sciences, RIKEN Center for Integrative Medical Sciences, Kanagawa, 230-0045, Japan

[5]Department of Microbiology and Immunology, Keio University School of Medicine, Tokyo, 108-8345, Japan

[6]Graduate School of Frontier Sciences, The University of Tokyo, Chiba, 277-8561, Japan

[7]Department of Gastroenterology and Hepatology, Tokyo Medical and Dental University, Tokyo, 113-8510, Japan

[8]Children's Center for Health and Development, Saiseikai Yokohamashi Tobu Hospital, Kanagawa, 230-8765, Japan

[9]Graduate School of Environmental and Life Science, Okayama University, Okayama, 700-0082, Japan

[10]Graduate School of Advanced Science and Engineering, Waseda University, Tokyo, 169-8555, Japan

[†]**Joint last co-authorship:** Tsuyoshi Sogo and Masahira Hattori contributed equally to this paper.

***Corresponding author**

Kentaro Iwasawa M.D.

Department of Pediatrics, Yokohama Minami Kyousai Hospital

1-21-1 Mutsuurahigashi, Kanazawa-ku, Yokohama, Kanagawa 236-0037, Japan

Tel: +81-45-782-2101, E-mail: ken.iwasawa@gmail.com

Supplementary Table S1. Statistics of the 16S pyrosequencing of salivary microbiota samples

Total reads	Filter-passed reads	Reads removed		
		Reads lacking primer sequences	Reads with average Qv<25	Possible chimeric reads
816,625<br>(100)	458,021<br>(56.1)	353,102<br>(43.2)	2,632<br>(0.3)	2,870<br>(0.4)

The numbers in parentheses indicate the ratio in percentage to the total read number.

Supplementary Table S2. Demographics and characteristics of PSC patients with and without SASP

	SASP (+)	SASP(−)	*p*-value
No. of patients	13	11	
Male, n (%)	7 (54)	9 (82)	0.211
Present age, yrs, median (IQR)	11 (10–13)	17 (8.5–18.5)	0.222
Age at onset, yrs, median (IQR)	6 (4–9)	8 (3–10)	0.720
Age at diagnosis, yrs, median (IQR)	9 (6–12)	9 (6.5–14.5)	0.577
PSC phenotype n (%)			
Large duct PSC	13 (100)	10 (91)	0.458
Smal duct PSC	0 (0)	1 (4)	0.458
Overlap with autoimmune hepatitis	9 (69)	5 (45)	0.408
Type of IBD, n (%)	13 (100)	11 (100)	1.000
UC	4 (31)	6 (46)	0.408
IBD-U	9 (69)	5 (53)	0.408
PUCAI score in UC patients, n (%)			
Remission (0- 9)	3 (75)	4 (67)	1.000
Mild (10-30)	1 (25)	2 (33)	1.000
Moderate to severe (35-)	0 (0)	0 (0)	1.000
Biochemical data, median (IQR)			
Platlets, $10^9/\mu L$	244 (224–288)	307 (274.5–390.5)	**0.039**
Albumin, g/dL	4.6 (4.2–4.8)	4.2 (3.75–4.5)	0.121
AST, IU/L	55 (24–86)	57 (29.5–77)	0.943
ALT, IU/L	36 (16–79)	50 (20–95.5)	0.680
GGT, IU/L	43 (12–64)	75 (36.5–232.5)	0.137
APRI	0.42 (0.22–1.42)	0.42 (0.24–0.54)	0.531
Medication, n (%)			
UDCA	13 (100)	7 (64)	**0.031**
Mesalazine	0 (0)	5 (45)	**0.011**
Immunosuppresive	7 (54)	2 (18)	0.105
Steroids	5 (38)	1 (9)	0.166
Probiotics	2 (15)	3 (27)	0.630

ALT, alanine aminotransferase; AST. asparate aminotransferase; APRI, AST to platlet ratio; GGT, γ-glutamyl transferase; HC, healthy control; IBD, inflammatory bowel disease; IBD-U, inflammatory bowel disease unclassified; IQR, interquartile range; PSC, primary sclerosing cholangitis; PUCAI, paediatric ulcerative colitis activity index; SASP, salazosulfapyridine; UC, ulcerative colitis; UDCA, ursodeoxycholic acid.

Supplementary Table S3. Evaluation of the effects of medication and phenotype on the overall structure of salivary microbiota in the PSC patients

Category	No. of subjects	Weighted UniFrac		Unweighted UniFrac	
		R^2	*p*-value	R^2	*p*-value
Overlap with AIH vs. no overlap with AIH	AIH: 14<br>no AIH: 10	0.04728	0.3027	0.0461	0.5155
UC vs. IBD-U	UC: 10<br>IBD-U: 14	0.01675	0.9870	0.03412	0.9371
SASP vs. no SASP	SASP: 13<br>no SASP: 11	0.02490	0.8811	0.03531	0.8621
UCDA vs. no UCDA	UCDA: 20<br>no UCDA: 4	0.05018	0.2787	0.06093	0.06593
Mesalazine vs. no Mesalazine	Mesalazine: 5<br>no Mesalazine: 19	0.01979	0.9690	0.03479	0.8611
Probiotics vs. no Probiotics	Probiotics: 5<br>no Probiotics: 19	0.05986	0.1708	0.05867	0.08092

Significant p-values are in bold. AIH, autoimmune hepatitis; IBD-U, inflammatory bowel disease unclassified; SASP, salazosulfapyridine; UDCA, ursodeoxycholic acid.

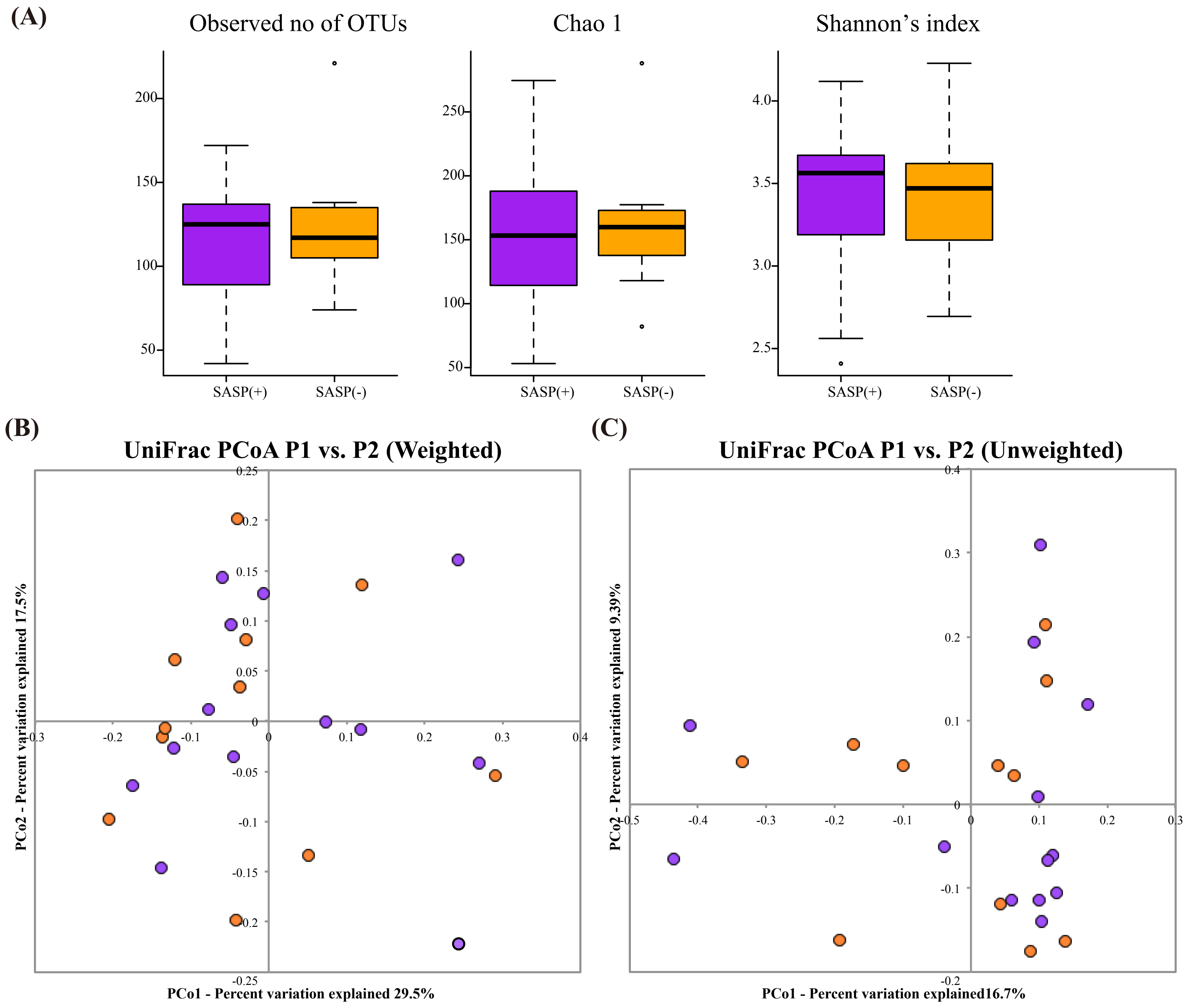

Supplementary Fig. S1. Comparison between the PSC patients treated with SASP and those not treated with SASP.
(A) The observed and Chao 1-estimated OTU numbers and the Shannon's index of salivary microbiota from the three groups. (B) Weighted UniFrac-PCoA and (C) unweighted UniFrac-PCoA of salivary microbiota from the SASP-treated (purple) and untreated (orange) samples. No significant difference was observed by the Kruskal-Wallis test between the SASP-treated and untreated groups. SASP, salazosulfapyridine; PCoA, principle coordinate analysis.

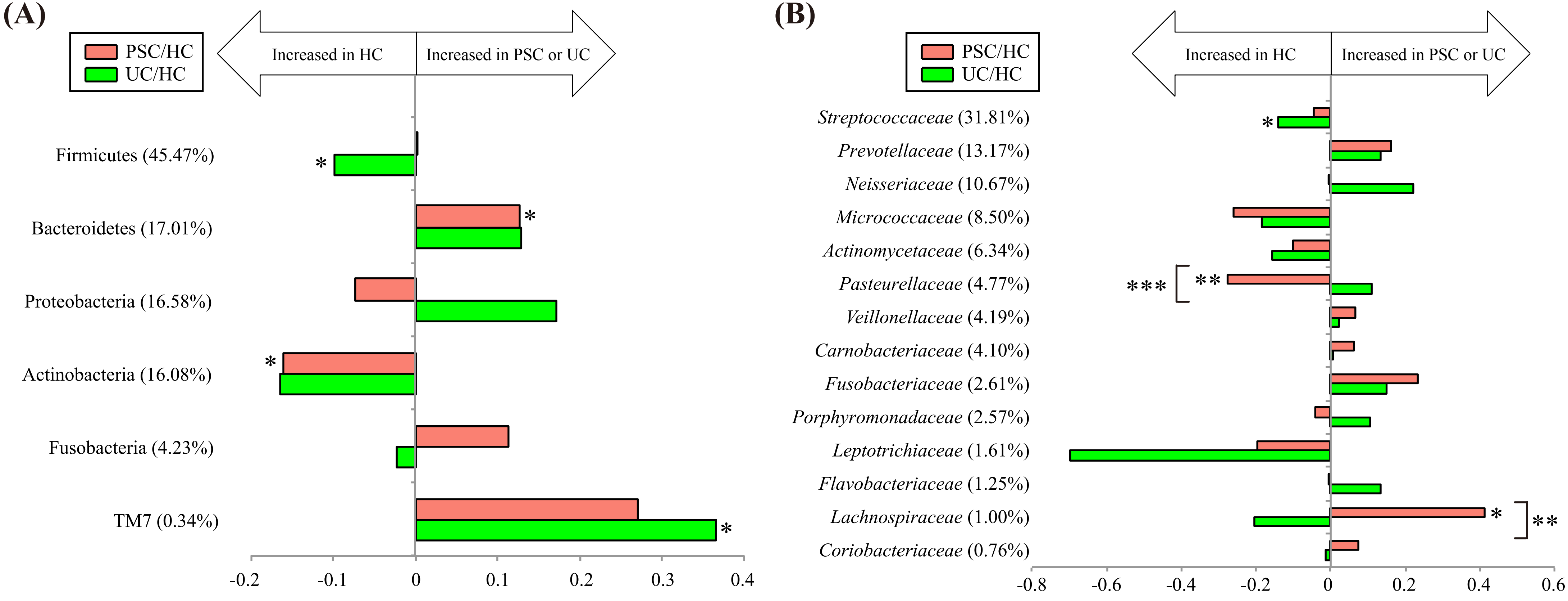

Supplementary Fig. S2. Comparison of the abundance of phyla and families among the PSC, UC, and HC subjects.
(A) The fold-change of six dominant phyla between PSC and HC, and between UC and HC samples. (B) The fold-change of 14 dominant families between PSC and HC and between UC and HC samples. The fold-change was calculated by dividing the mean relative abundance of each phylum and family in the PSC and UC groups by that in the HC group, respectively. The mean abundance (%) in the HC samples is shown in parentheses. Horizontal axis: The fold-change displayed in log10. Horizontal bars indicate fold-change between PSC and HC (red) and between UC and HC (green). *p<0.05, **p<0.01, ***p<0.001 based on the Kruskal-Wallis test followed by the Steel-Dwass test for multiple comparisons.

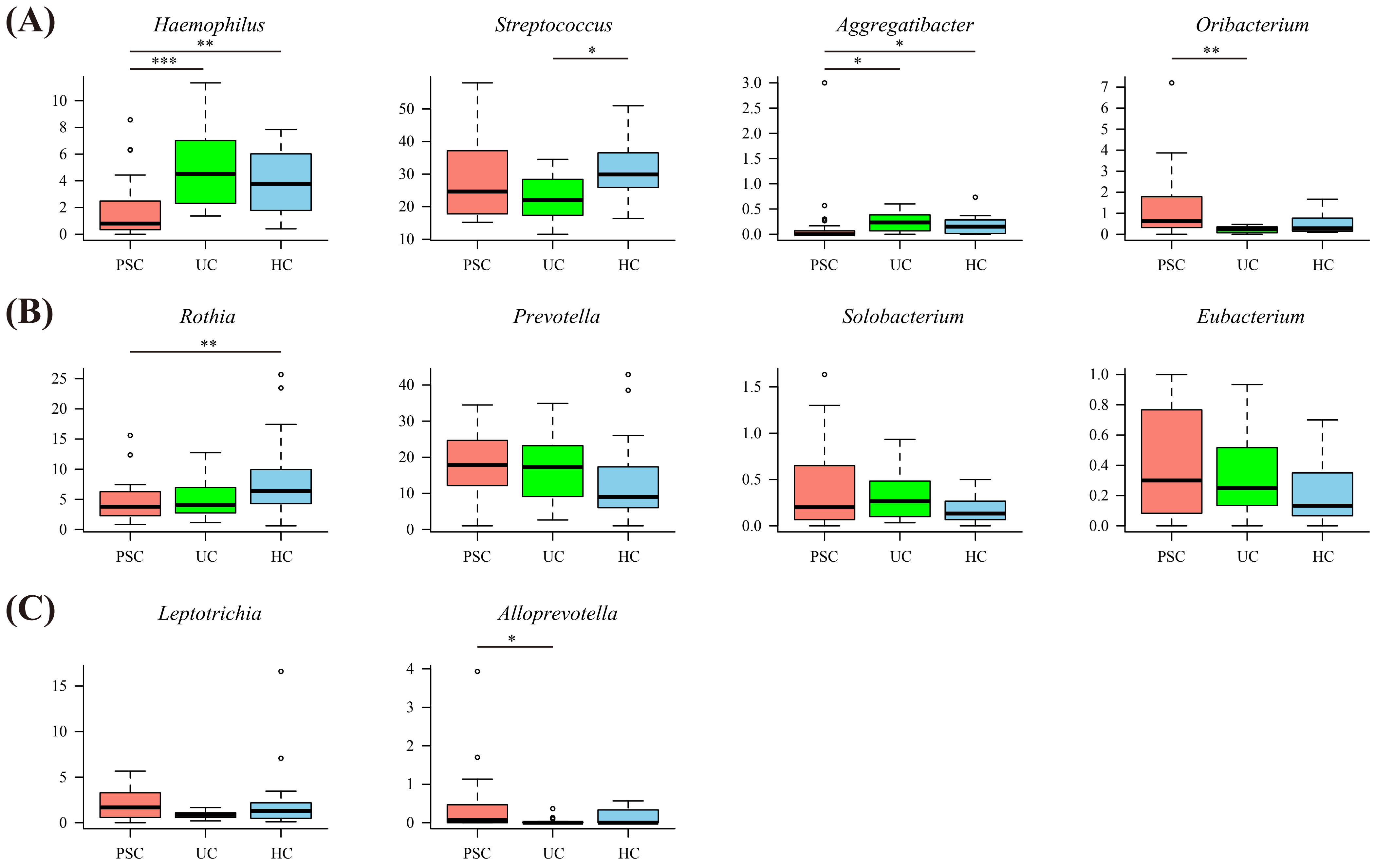

Supplementary Fig. S3. Comparison of abundance of the selected genera in the area under the curve-random forest (AUC-RF). Box plot of the relative abundance of the 10 selected genera in AUC-RF. Genera that contributed to distinguish (A) the PSC from both the HC and UC groups, (B) the PSC from the HC group, and (C) the PSC from the UC group. *$p<0.05$, **$p<0.01$, ***$p<0.001$ based on the Kruskal-Wallis test followed by the Steel-Dwass test for multiple comparisons.

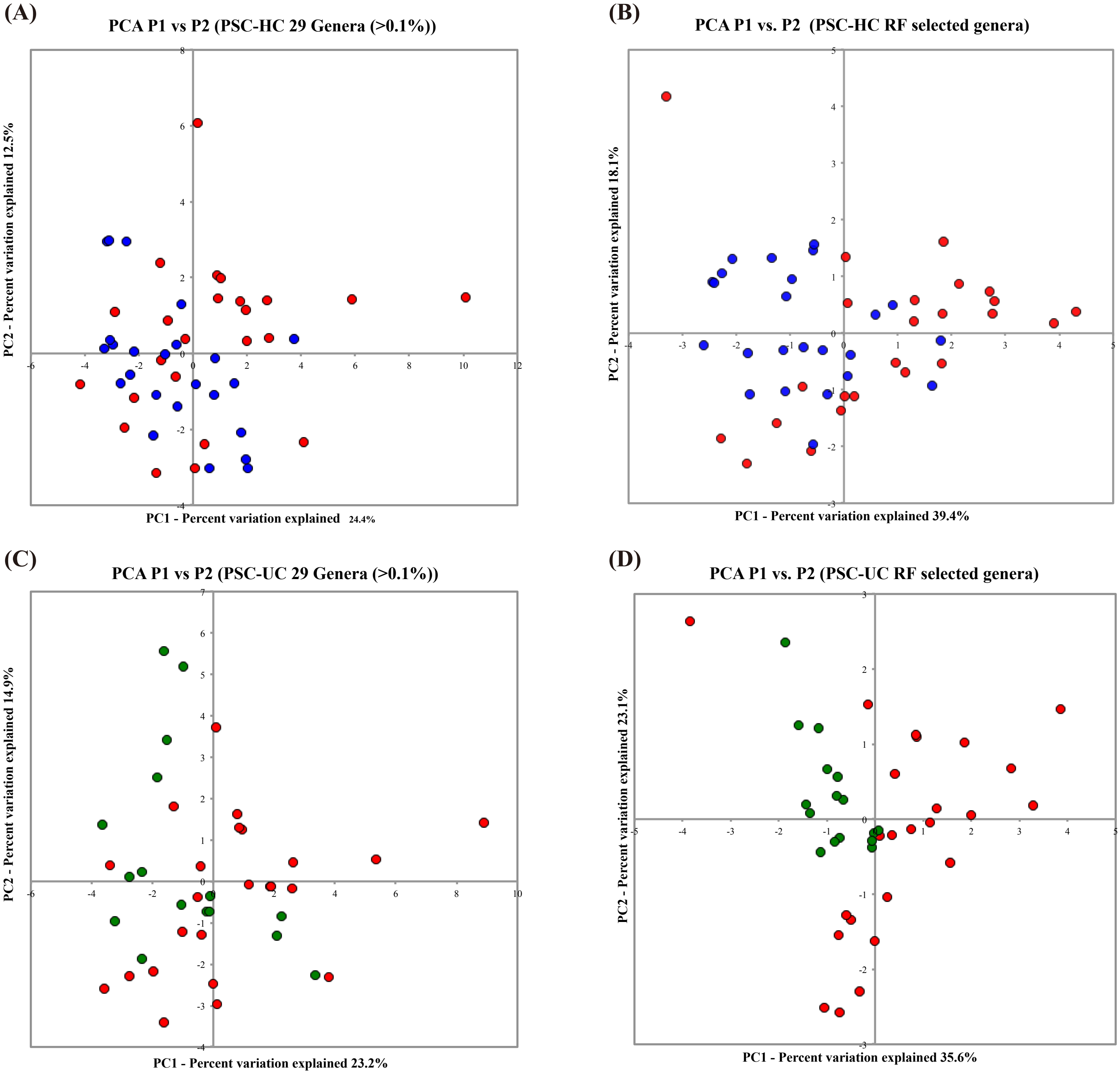

Supplementary Fig. S4. PCA of the selected genera in AUC-RF.

(A) The PCA based on the abundance of the 29 abundant genera with relative mean abundances of >0.1% and (B) the PCA based on the abundance of the RF-selected genera between PSC and HC. Similarly, (C) the PCA of the 29 genera and (D) the RF-selected genera between the PSC and UC groups. PSC (red), UC (green), and HC (blue) samples are shown. PCA, principal component analysis.

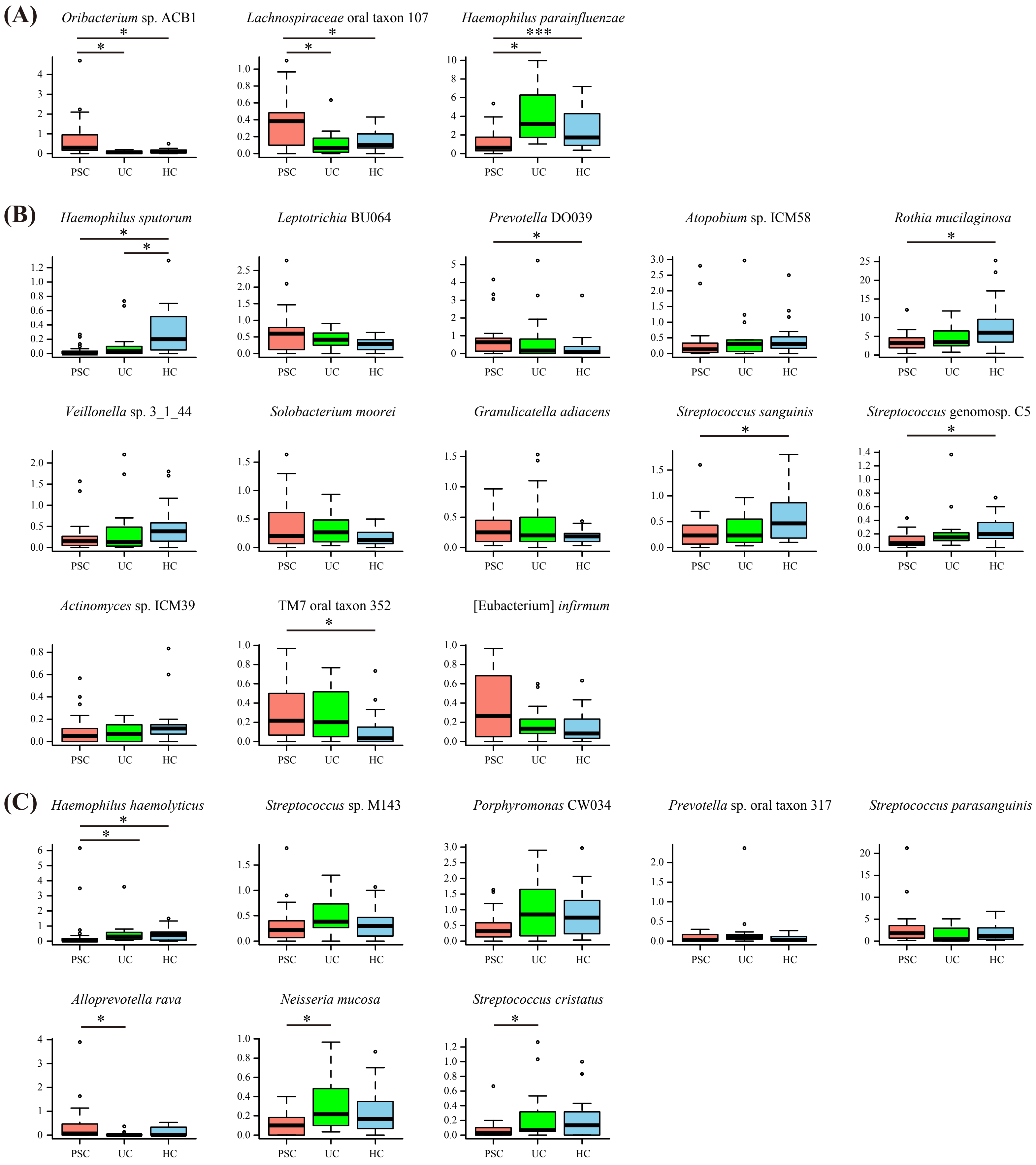

Supplementary Fig. S5. Comparison of the abundance of the selected species in AUC-RF. Box plot of the relative abundance of the 24 selected species in AUC-RF. Species that contributed to distinguish (A) the PSC from both the HC and UC groups, (B) the PSC from the HC group, and (C) the PSC from the UC group. $*p<0.05$, $**p<0.01$, $***p<0.001$ based on the Kruskal-Wallis test followed by the Steel-Dwass test for multiple comparisons.

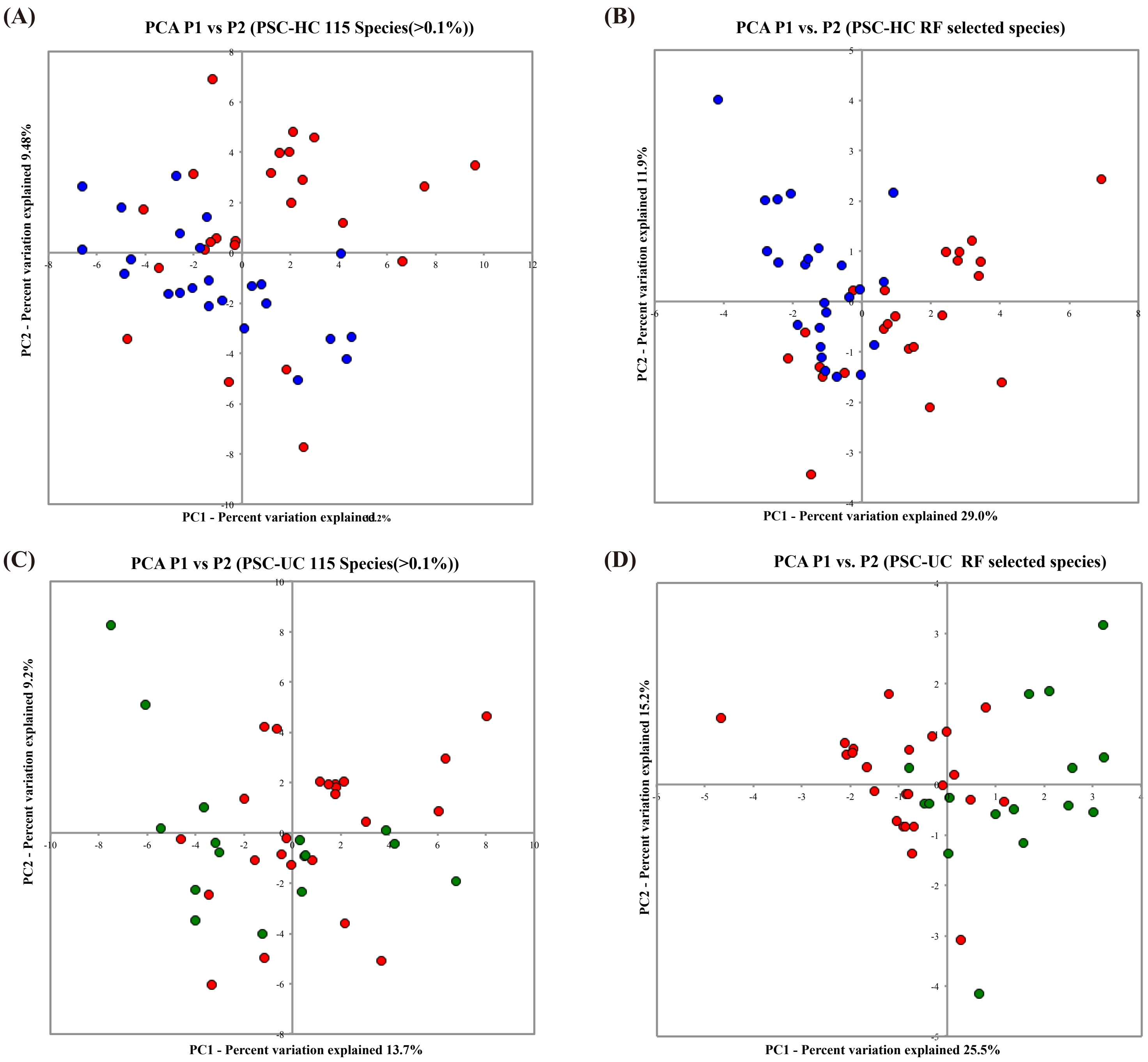

Supplementary Fig. S6. PCA of the selected species in AUC-RF.

(A) The PCA based on the abundance of the 95 abundant species with relative mean abundances of >0.1% and (B) the PCA based on the abundance of the RF-selected species between PSC and HC. Similarly, (C) the PCA of the 95 species and (D) the RF-selected species between the PSC and UC groups. PSC (red), UC (green), and HC (blue) samples are shown.

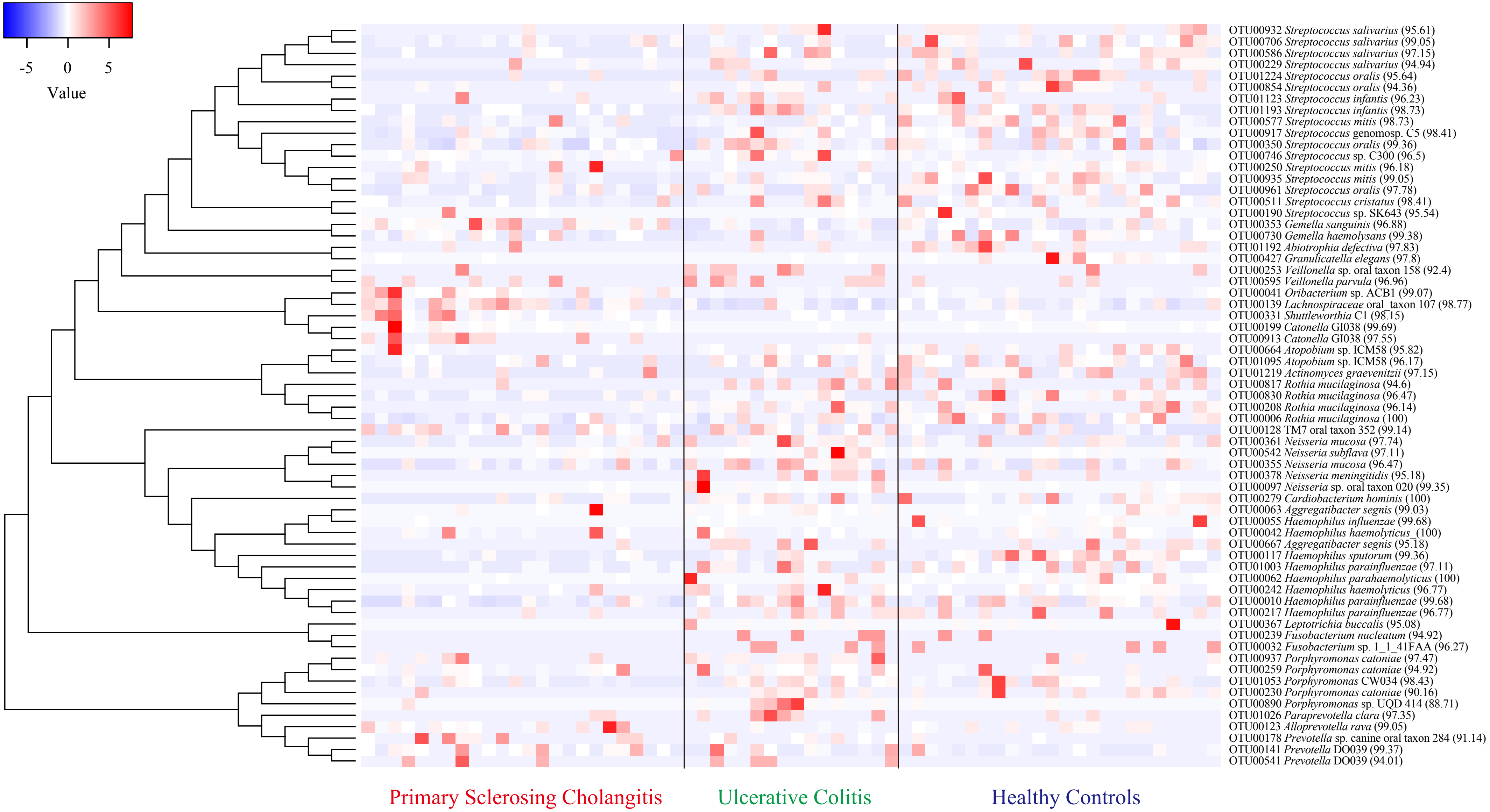

Supplementary Fig. S7. Heatmap of the abundances of 65 OTUs in PSC, UC, and HC samples.
The OTUs exhibiting significant differences ($p<0.05$) among the three groups are shown. Statistical significance was evaluated by the Kruskal-Wallis test followed by the Steel-Dwass test for multiple comparisons. Colors represent Z-scores. A phylogenetic tree was constructed based on the sequences of the OTUs and is shown on the left side of the heatmap. The OTU number and assigned species are shown on the right side of the heatmap.
